# The serum uric acid is longitudinally related to patients global assessment of disease activity in male patients with axial spondyloarthritis

**DOI:** 10.1186/s12891-022-05657-3

**Published:** 2022-07-27

**Authors:** Meimei Cai, Wen Liu, Yuanhui Wu, Qing Zheng, Dehao Liu, Guixiu Shi

**Affiliations:** 1 Department of Rheumatology and Clinical Immunology, The First Affiliated Hospital of Xiamen University, School of Medicine, Xiamen University, Xiamen, Fujian China; 2 Department of Rheumatology and Clinical Immunology, Jiangxi Provincial People’s Hospital Affiliated of Nanchang University, Nanchang, China; 3 Department of Rheumatology and Clinical Immunology, The First Affiliated Hospital of Fujian Medical University, Fuzhou, China; 4 Department of Radiology, The First Affiliated Hospital of Xiamen University, School of Medicine, Xiamen University, Xiamen, Fujian China

**Keywords:** Axial spondyloarthritis, Serum Uric acid, Patient global assessment, SF-36, Longitudinally

## Abstract

**Objectives:**

To investigate longitudinal relationship between serum uric acid (SUA) and disease activity among Chinese males with axial spondyloarthritis (axSpA).

**Methods:**

Two-year data from the NASA study cohort of male patients with axial spondyloarthritis were analyzed. Patients global assessment of disease activity (PtGA), BASDAI, ASDAS-CRP, BASFI, and SF-36 were used as the outcomes. The autoregressive Generalized Estimation Equation (GEE) model was used to investigate the longitudinal relationship between SUA and the above outcomes. Age and gender and symptom duration were tested as effect modifiers or confounders.

**Results:**

In total, 102 male axSpA patients were included, 33.3% of who were hyperuricemia at baseline. Over time,serum uric acid levels associated with the global assessment of patient global assessment of disease activity (PtGA)[*P*=0.041, β=-2.059,95%CI(-4.032, -0.086)], SF-36: Vitality (VT) [*P*=0.01, β=1.751, 95%CI (0.415,3.087)], SF-36: Social Functioning (SF)[*P*=0.002, β= 2.968,95%CI (1.067,4.869)]). And these relationgships were independent of age, symptom duration, baseline uric acid levels, and medication use.

**Conclusions:**

In summary, SUA levels is longitudinally related to PtGA and mental health assessment. Age, gender and symptom duration do not have an impact on the relationships.

**Supplementary Information:**

The online version contains supplementary material available at 10.1186/s12891-022-05657-3.

## Introduction

Axial spondyloarthritis (axSpA) is a chronic inflammatory rheumatic disorder, and the clinical manifestations are varied [1]. In general, axSpA mostly affects the spinal column and the sacroiliac joint [2], resulting in inflammatory back pain and progressive bony fusion [3]. Additionally, extraarticular manifestations affect the patient's prognosis and quality of life [4]. The ASAS/EULAR international guidelines recommend that clinicians need to be alert and pay attention to extra-articular manifestations, which can be beneficial in the treatment of axSpA. Common extra-articular manifestations of axSpA such as uveitis, psoriasis and intestinal inflammation have been reported, but there are limited studies related to the effect of SUA levels on axial sclerosis.

Uric acid is an end product of purine metabolism. As purines are among the primary components of DNA and RNA, Uric acid is closely associated with life. Accordingly, as living conditions have improved in China over the past two decades, increasing levels of SUA have been linked to excessive consumption of purine-rich foods (such as red meat and seafood), unhealthy lifestyle habits (such as smoking, excessive alcohol consumption, and sedentary habits), and underlying comorbidities [5, 6]. The study shown that low levels of serum uric acid have a significant negative effect on bone mineral density in axSpA patients [7]. In a cross-sectional study, it was found that patients with hyperuricemia had much lower levels of the condition compared to patients without hyperuricemia in China [8]. The study, however, was based on cross-sectional data analysis, which look at data collected over time, and the results do not offer a complete picture. AxSpA is a chronic inflammatory immune disease with a recurrent inflammatory course [9], so the cross-sectional study cannot reflect the effect of time changes on the disease and SUA levels.

The current studies of SUA in spondyloarthritis are cross-sectional. Despite this, little is known about the relationship between SUA and disease indicators over time. Disease activity measurement in axial SpA included patient global assessment (PtGA), spinal pain , BASDAI, ASDAS-CRP, and Health-related Quality of Life Score (HRQoLs). Our objective was to determine if SUA levels was longitudinally related to disease activity measures over time in patients with axSpA.

## Methods

### Study design and population

The effect of nonsteroidal anti-inflammatory drugs on relapse after remission in patients with spondyloarthritis (NASA study, Clinical Trials. gov ID: NCT03425812) is an ongoing multicenter, randomized, parallel controlled study being conducted in Fujian, China, from 2018 to 2021 The NASA study was designed to investigate relapse rates in patients with axial arthritis in remission who withdrew from nonsteroidal anti-inflammatory therapy. We build a single-center cohort study based on The NASA study, participants who met the following criteria were included in our study: age 18 years at screening; those who diagnosed as axSpA by rheumatologists according to the Assessment of Spondyloarthritis International Society (ASAS) classification criteria [10, 11]. Clinical data was collected every 3 months, Follow up for at least 2 years. Clinical data including vital signs, disease assessment, and laboratory tests (such as CRP and uric acid) Patients who had SUA tested at least twice during the two-year follow-up were the subject of this analysis. Other exclusion criteria were reactive arthritis, thyroid or parathyroid disease, chronic liver disease, and chronic kidney disease [glomerular filtration rate (GFR) <90 ml/min/1.73 m2]. Patients who have used allopurinol, fenofibrate, β-blockers, or diuretics in the past were also excluded due to their potential effect on serum UA levels. Patients who were included in NSAIDs elution phase and randomized grouping in NASA

study were also excluded.

Ethical approval for the NASA study, including the consent procedure, was granted by the Research Ethics committee of the First Affiliated Hospital of Xiamen University (reference number: KYH2018-007) with the Declaration of Helsinki V and the Danish legislation. Participants provided written informed consent prior to inclusion in the study.

### Outcome

Indicators used to describe disease activity: PtGA (collected on a 0-100 numeric scale by asking for an overall assessment of rheumatic disease activity in the previous week), self-reported spinal pain score (collected on a 0-100 numeric scale by asking for an assessment of overall spinal pain in the previous week and an assessment of nocturnal spinal pain), BASDAI (0-10) [12], ASDAS-CRP [13]，the BASFI (0-10) [14], erythrocyte sedimentation rate (ESR), C-reactive protein (CRP) level (mg/l), and HRQoLs was assessed by using a 36-item The Medical Outcomes Study Short-Form (SF-36) Questionnaire [15]. In the SF-36, eight dimensions of health are measured: physical functioning, bodily pain, role limitations due to physical health issues, role limitations due to personal or emotional issues, general mental health, social functioning, energy/fatigue, and overall health perceptions. Each sub-scale has a score ranging from 0 to 100. Better scores indicate a healthier state [16]. The outcomes were entered into the GEE model analysis as numerical variables.

### Contextual factors

The patient's age, Symptom duration, medication use, and baseline uric acid level were background factors that were tested as potential effect modifiers or confounders of the relationship between SUA determinants and Outcome. Men were considered hyperuricemic when their SUA levels were higher than 6.8 mg/dL (404.5 micromol/l) at baseline. Every follow-up visit included a medication usage record, consultation for NSAID use, and at least one conventional synthetic disease-modifying antirheumatic drug (cDMARD), and biologic agent (bDMARD). Dichotomize based on yes or no. If stratification for age, Symptom duration, medication use，or baseline uric acid level was needed, the population was dichotomized by age at baseline (younger or older than the median age at baseline, 33.3 years), Symptom duration (more than 10years or not), medication (use or not) or baseline uric acid level (hyperuricemic or not). These medication were considered noteworthy only if the patient related a history of regular administration of a particular therapy, and if the total time of the therapy administration (both regular administration and tapering) was greater than 50% of the time between each follow-up visit.

### Statistical analysis

To investigate the relationship between the SUA and outcomes, we used generalized estimating equations (GEEs). This method enabled us to make use of all available data and estimate a population-averaged parameter, correcting for within-patient correlation of outcomes at multiple time points. A linear GEE model was used because the outcome was continuous. An exchangeable correlation matrix was selected because it showed the best fit.

Interactions with age, Symptom duration, medication use，or baseline uric acid level were tested. If relevant interactions were found, analyses were stratified. If not, variables were entered as covariates.

As relationships found in GEE models can be attributable both to cross-sectional and longitudinal effects, an autoregressive GEE model (i.e., a model adjusted for the outcome at the previous time point) was used to investigate whether the SUA had a true longitudinal association with outcome.

Univariable and multivariable analyses were undertaken. The context factors were found to have no interaction in the model and were therefore included in the multivariable analysis as confounding factors.. SPSS 26 was used in all analyses.

## Result

In total, 102 patients were included in the study. In the present study, the average age was 35.54 (8.69) years, and the mean duration of disease was 11.25 years. All patients met the inclusion criteria and were followed for more than 2 years. However, 72 patients had SUA results at the second year with a baseline of 6.62 ± 1.52 mg/dl, 33.33% were hyperuricemic, their baseline socio-demographic and clinical characteristics are summarised in table 1. In the baseline clinical data of patients with hyperuric acid and normal uric acid levels, there was no significant difference (Table 2).

**Table 1:** Baseline characteristics of the study patients (*n=* 102 at baseline, *n=* 75 at 2 years))

Independent variable	Baseline	2 years
Age	35.54±8.69	–
erum uric acid,mg/dl	6.62±1.52	6.39±1.39
hyperuricemia no. (%)	34 (33.33%)	22 (29.33%)
HLA–B27 positive, no. (%)	85 (83.33)	–
Symptom duration, years	11.25±8.15	–
History of uveitis, no. (%)	11 (10.78)	–
History of psoriasis, no. (%)	0 (0)	–
History of IBD, no. (%)	2 (1.96)	–
History of peripheral arthritis, no. (%)	16 (15.68)	–
csDMARDs use, no. (%)	60 (58.82)	–
NSAID use, no. (%)	59 (57.84)	–
TNFi use, no. (%)	23 (22.55)	–
**Outcome**		
PtGA,0–100	17.86±18.82	13.01±15.26
Spinal pain ,0–100	15.09±15.29	12.37±18.1
Spinal pain in ninght,0–100	13.34±16.18	11.09±17.78
BASDAI,0–10	1.41±1.29	1.06±0.96
BASFI,0–10	0.61±0.96	0.43±0.69
ASDAS-CRP	1.31±0.75	1.37±0.67
SF-36:PF (Physical Functioning)	87.64±14.46	91.66±10.42
SF-36:RP (Role-Physical)	69.85±40.77	77.45±38.48
SF-36:BP (Bodily Pain)	72.66±13.58	75.92±14.04
SF-36:GH (General Health)	57.3±19.31	60.94±21.41
SF-36:VT (Vitality)	65.14±13.99	62.64±10.59
SF-36:SF (Social Functioning)	89.46±18	89.7±20.57
SF-36:RE (Role-Emotional)	73.52±38.2	79.73±35.94
SF-36:MH (Mental Health)	50.5±14.03	61.56±12.75
CRP (mg/liter)	5.02±6.59	6.56±7.31
ESR (mm/h)	10.18±10.35	8.95±7.3

Except where indicated otherwise, values are the mean ± SD

*PtGA* Patient global assessment of disease activity, *ASDAS-CRP* Ankylosing Spondylitis Disease Activity Score using the C-reactive protein level, *BASDAI* Bath Ankylosing Spondylitis Disease Activity Index, *BASFI* Bath Ankylosing Spondylitis Functional Index, *IBD* Inflammatory bowel disease, *NSAID* Nonsteroidal antiinflammatory drug, *TNFi* Tumor necrosis factor inhibitor

**Table 2:** Baseline characteristics between Normal uric acid group and hyperiuricemia

Independent variable	Normal uric acid group	hyperuricemia group	P
Number	68	34	-
Age	36.6±9.02	33.44±7.67	0.078
rum uric acid,mg/dl	5.79±0.97	8.27±0.97	-
HLA–B27 positive, no. (%)	63 (92.65)	32 (94.12)	0.782
Symptom duration, years	11.42±8.89	10.91±6.54	0.757
History of uveitis, no. (%)	8 (11.76)	3 (8.82)	0.652
History of psoriasis, no. (%)	0 (0)	0 (0)	-
History of IBD, no. (%)	2 (1.96)	0 (0)	-
History of peripheral arthritis, no. (%)	11 (16.17)	5 (14.71)	0.8
csDMARDs use, no. (%)	41 (64.06)	19 (55.89)	0.67
NSAID use, no. (%)	38 (59.375)	21 (61.18)	0.57
TNFi use, no. (%)	15 (22.06)	8 (23.53)	
**outcome**			
PtGA，0–100	20.02±20	13.52±15.58	0.075
Spinal pain ,0–100	16.69±16.1	11.91±13.18	0.114
Spinal pain in ninght,0–100	14.82±16.85	10.38±14.54	0.173
BASDAI,0–10	1.58±1.42	1.07±0.92	0.031
BASFI,0–10	0.63±0.9	0.55±1.08	0.692
ASDAS-CRP	1.38±0.79	1.17±0.65	0.164
SF-36:PF (Physical Functioning)	87.27±14.62	88.38±14.34	0.523
SF-36:RP (Role-Physical)	74.26±38.86	63.23±43.62	0.206
SF-36:BP (Bodily Pain)	72.63±14.11	74.17±12.87	0.421
SF-36:GH (General Health)	58.3±19.44	56.32±18.17	0.527
SF-36:VT (Vitality)	67.13±12.67	60.14±15.19	0.019
SF-36:SF (Social Functioning)	89.33±18.35	90.44±18.47	0.74
SF-36:RE (Role-Emotional)	73.52±38	72.54±38.89	0.891
SF-36:MH (Mental Health)	50.35±13.99	50.47±14.43	0.853
CRP (mg/liter)	5.26±7.24	4.54±5.13	0.763
ESR (mm/h)	9.71±10.75	11.08±9.62	0.16

Except where indicated otherwise, values are the mean ± SD

*PtGA* Patient global assessment of disease activity, *ASDAS-CRP* Ankylosing Spondylitis Disease Activity Score using the C-reactive protein level, *BASDAI* Bath Ankylosing Spondylitis Disease Activity Index, *BASFI* Bath Ankylosing Spondylitis Functional Index, *IBD* Inflammatory bowel disease, *NSAID* Nonsteroidal antiinflammatory drug, *TNFi* Tumor necrosis factor inhibitor

**Fig. 1:**
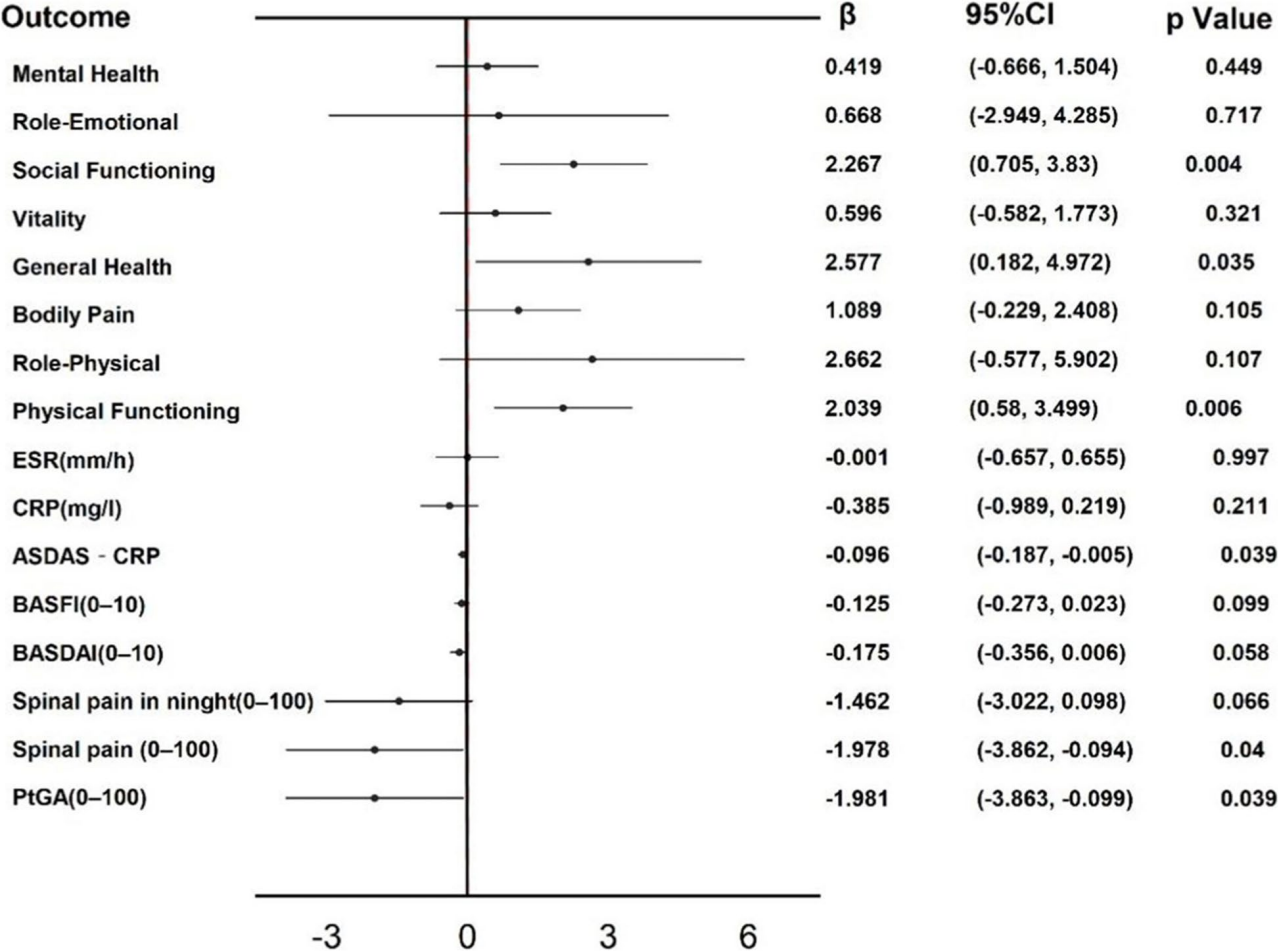
Longitudinal relationship between SUA and the outcome indexs during the follow-up in male patients with axial spondyloarthritis (Univariable Model)

In the univariable generalized estimating equations (GEE) model, ,SUA levels as continuous variables, outcomes as the dependent variables, SUA was negatively correlated with PtGA in univariable analysis [*P*=0.039, β=-1.9981, 95%CI (-3.863, -0.099)] and spinal pain [*P*=0.04,β=-1.9979, 95%CI (-3.862, -0.094)], On the other hand, ASDAS-CRP, BADAI, BASFI, CRP, and ESR did not show a significant correlation with SUA (Figure 1, Table S1). Based on the univariable analysis that analyzed the relationship between SUA levels and health-related quality of life (HRQoL), it was found that SUA levels had a positive relationship with Physical Functioning (PF) [*P*=0.006, β=2.039, 95%CI (0.58, 3.499)], General Health (GH) [*P*=0.035, β=2.577, 95%CI (0.182, 4.972)], and Social Functioning (SF) [*P*=0.004, β=2.267, 95%CI (0.705, 3.83)]. No significant correlation was seen with other SF-36 indicators (Figure 1, Table S1).

**Fig. 2:**
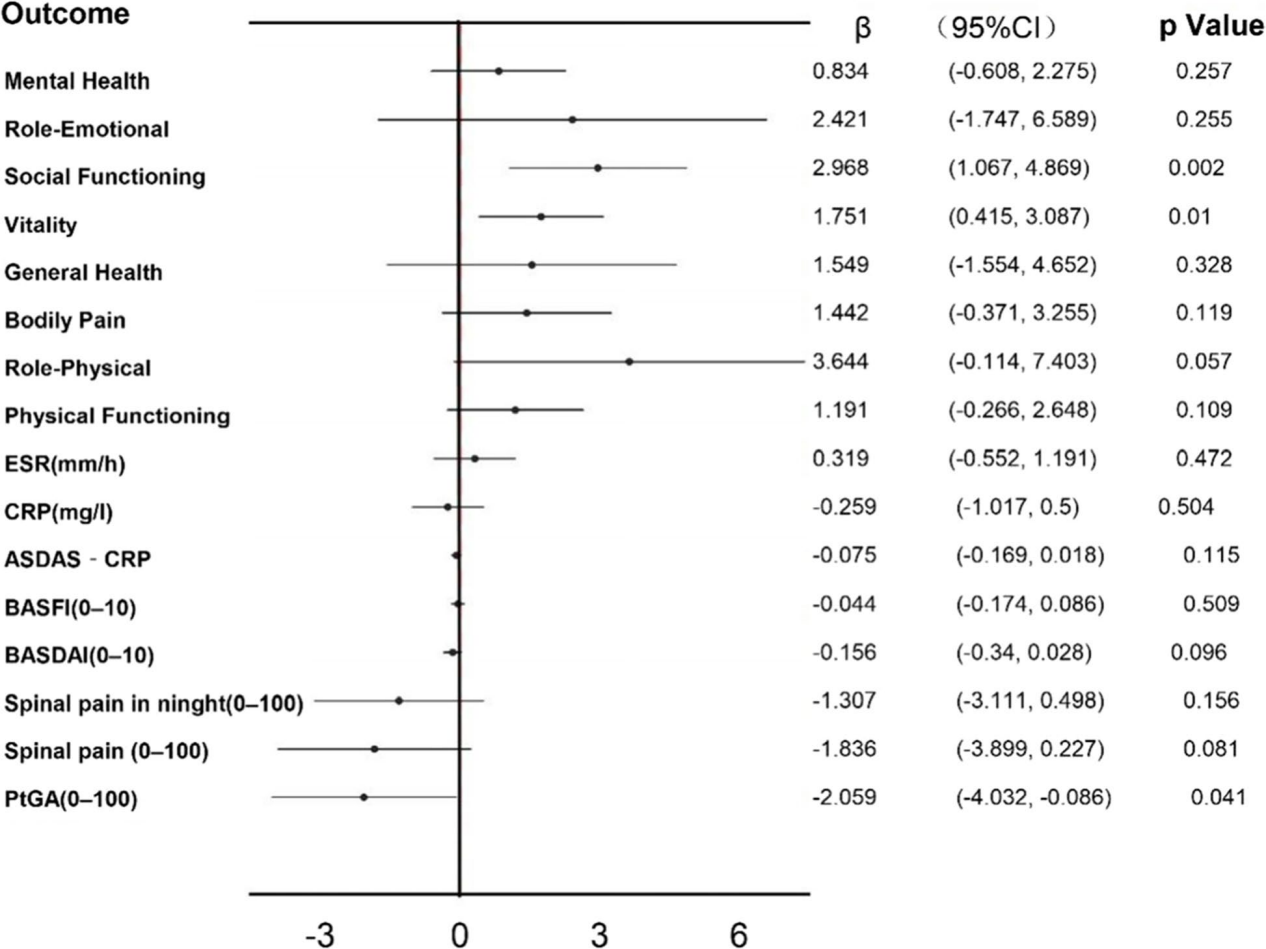
Longitudinal relationship between SUA and the outcome indexs s during the follow-up in male patients with axial spondyloarthritis (Multivariable Model Adjusted for Contextual factors )

Contextual factors were not effect modifiers of the relationship between the PtGA and SUA (Supplementary Table S3–S6), so we did not perform a stratified analysis. Multivariable generalized estimating equations (GEE) were used to eliminate the influence of confounding factors. The results indicate that SUA level is negatively related to PtGA [*P*=0.041, β=-2.059,95%CI(-4.032, -0.086)]. This effect was independent of age, symptom duration, hyperuricemia at baseline, and medication use, No relationship was found between spinal pain and SUA in the multivariable analysis, and no relationships were seen with SUA for other indicators of disease activity (Figure 2, Table S2). In multivariable model analysis with SF-36, SUA was still positively correlated with Vitality (VT) [*P*=0.01, β (95% confidence interval) and Social Functioning (SF) [*P*=0.002, β=2.968,95%CI (1.067,4.869)], indicating that the quality of life of patients with higher SUA was improved. However there is a significant interaction between SF and Medication use, since the number of cases was small, we did not perform stratified analysis in these cases (Figure 2, Table S2).

## Discussion

To the best of our knowledge, it is the first time to explore the effect of SUA levels on axSpA from longitudinal cohort data. UA is known to function as an important oxidant in the human body [17], Hyperuricosuria is harmful to human health [18]. But is it seems to indeed be the case in axSpA patient? The longitudinal observation of SUAand axSpA disease has not been reported. we used a longitudinal observational cohort study for the first time to study the effect of SUA levels over time in patients with axSpA. In the results of this study, we observed a longitudinal relationship between SUA levels and PtGA and mental health assessment. Exploring the relationship between SUA levels and disease activity in axSpA patients over time suggests that hight SUA levels during treatment has the negative relationship with PtGA in axSpA. We use the univariable GEE analysis in this study, the results suggested that during follow-up, a decrease in PtGA with increasing SUA level, and the same with spinal pain, contextual factors were included in the multivariable analysis model, this relationship was statistically significant.

In our cohort study, the longitudinal relationship between SUA levels and PtGA was independent of medication use. The results can be seen in (Tables S3-S6). Serum creatinine levels, urea nitrogen, and other biochemical indexes in the patient remained stable and no side effects were observed during the follow-up. Therefore, we conjectured that renal dysfunction and drugs were not the cause for higher levels of SUA in our cohort study, which might due to the remision of inflammatory. As a result of the analysis, It was found that the patient had increased SUA levels and decreased PtGA levels.

During clinical observation, the SUA levels of gout patients in the acute phase were within normal value s[19]. Waldron and colleagues demonstrated a significant reduction in SUA when patients without gout during a systemic inflammatory response (SIR) provoked by orthopedic surgery [20]. In recent years, some scholars have proposed decreasing systemic inflammation leads to an increase in serum urate [21]. For example, people found an increased level of SUA that patients after treated with three mouths anti-TNF inhibitors in a study from 201 9[22]. It seems that uric acid elevation is associated with decreased levels of inflammation in axSpA.

Uric acid is the end-product of endogenous and dietary purine metabolism [23]. In addition to renal excretion, elevated uric acid is related to the endogenous purine metabolism. Hence the metabolic rate of endogenous purine metabolism maybe cause more adenosine. Extracellular adenosine exerts immunosuppressive effects on immune cells via the adenosine receptor [24], resulting in the reduced production and release of inflammatory cytokines [24]. In fact, there is a growing body of evidence supporting the concept that regulatory T cells suppress effector T cells mainly via generating adenosine [25, 26]. In preclinical studies, adenosine A2_A_ receptor agonists have a wide range of anti-inflammatory effects in inflammatory diseases. In the preclinical models of rheumatoid arthritis [27], encephalomyelitis [28] andallergic asthma [29] beneficial effects of A2_A_ receptor agonists have been observed. Adenosine receptors are associated with the remission of the disease among AS patients [30]. It is suggested that adenosine receptors are involved in reducing inflammation of AS patients [31], the anti-inflammatory effects of disease-modifying antirheumatic drugs approved for the clinical treatment of axSpA is also associated with adenosine receptors. For instance, Adenosine mediates the anti-inflammatory effects of methotrexate [32]. The A2_A_ receptor is the receptor responsible for the anti-inflammatory effects of sulfasalazine [33]. It is, of course, possible that adenosine receptors and uric acid corporately participate in the regulation of inflammatory responses. In a mouse model of colitis, the efficacy of adenosine receptor agonists in relieving enteritis might be more pronounced with a higher level of uric acid [34]. Therefore, we speculated that the remission of auto-inflammation may account for the enhanced uric acid levels. The potential mechanism may be related to the metabolism of adenosine.

SUA is association with SF36 scores (8 domains) was considered during data analysis, and it was positively and significantly correlated to Social Functioning and Vitality (the scores in the SF-36 can range from 0 to 100), while higher scores indicate better health status. The 8 multi-item scales of the SF-36 were summarized into two components (mental and physical components respectively) [35]. Social Functioning and Vitality belonged to the SF36 mental health domain [35], our data suggested that SUA improved the SF36 mental health domain scores. Maybe improvement in mental health associated with the amelioration of inflammatory. But the effect of uric acids in mental cannot be ruled out. Early observations in manic patients reported an increased excretion of uric acid [36], later studies supported SUA levels are associated with mania and depression [37]. Experimental studies supported that different adenosine concentrations and adenosine receptors had anti-depressant or anti-anxiety effects [38]. So uric acid as a metabolite of adenosine might have an effect on mental. However, the specific mechanism is unclear.

## Conclusions

We find that SUA levels is longitudinally related to PtGA and mental health assessment Age, gender and symptom duration do not have an impact on the relationships. during the follow-up period. No significant association with inflammation has been found. In the light of the complexity of the pathogenesis of axSpA, the association of subjective symptoms remission with SUA level maybe is part of the pathogenesis of axSpA. Uric acid is the final product of adenosine metabolism. We suggest adenosine and adenosine receptors may play important roles in the pathogenesis of axSpA. And further studies are warranted to investigate their relationship and the underlying mechanism.

The limitations of this study were summarized. Firstly, the follow-up period was short and the sample size was also limited. The patients enrolled in this study may not be representative of all axSpA patients. Secondly, the patients taking urate-lowering therapy within 3 months before into the group were excluded, and the effect of therapy on hyperuricemia could not be analyzed.

### Significance and Innovation

1. This is the first longitudinal observational cohort study of the effect of SUA **l**evels over time on axSpA patients.
2. In the longitudinal cohort study, we observed a significant association between SUA levels and remission of PtGA and mental health assessment.

## Supplementary Information

**Additional file 1: Table S1.** Longitudinal relationship between serum uric acid and the outcome indexs during the follow-up in male patients with axial spondyloarthritis (Univariable Model). **Table S2.** Longitudinal relationship between SUA and the outcome indexs s during the follow-up in male patients with axial spondyloarthritis (Multivariable Model Adjusted for Contextual factors ). **Table S3.** Effects of hyperuricemia on the outcome indexs during the fllow-up in male patients with axial spondyloarthritis (Multivariable Model Adjusted for Contextual factors ). **Table S4.** Effects of bDMARD use on the outcome indexs during the fllow-up in male patients with axial spondyloarthritis (Multivariable Model Adjusted for Contextual factors ). **Table S5.** Effects of cDMARD use on the outcome indexs during the fllow-up in male patients with axial spondyloarthritis (Multivariable Model Adjusted for Contextual factors ). **Table S6.** Effects of NSAID use on the outcome indexs during the fllow-up in male patients with axial spondyloarthritis (Multivariable Model Adjusted for Contextual factors).

## Data Availability

The data set are not publicly available but are available from the corresponding author on reasonable request.

## Abbreviations

AxSpA: Axial spondyloarthritis
PtGA: Patients global assessment of disease activity
ASAS: International Society for the Assessment of Spondyloarthritis
BASDAI: Bath Ankylosing Spondylitis Functional Index
ASDAS-CRP: Ankylosing Spondylitis Disease Activity Score
BASFI: GEE Generalized Estimation Equation
IBP: Inflammatory back pain
SUA: Serum uric acid
cDMAR: Conventional synthetic disease-modifying antirheumatic drug
bDMARD: Biologic disease-modifying antirheumatic drug
HRQoL: Health-related quality of life
PF: Physical Functioning
GH: General Health
SF: Social Functioning
SIR: systemic inflammatory response
