# Supplementary material for: The serum uric acid is longitudinally related to patients global assessment of disease activity in male patients with axial spondyloarthritis
Source: BMC Musculoskelet Disord. 2022 Jul 27;23:717. doi: 10.1186/s12891-022-05657-3 (PMC9327298; doi:10.1186/s12891-022-05657-3)
Supplement: Supplementary file 1 — Additional file 1: Table S1. Longitudinal relationship between serum uric acid and the outcome indexs during the follow-up in male patients with axial spondyloarthritis (Univariable Model). Table S2. Longitudinal relationship between SUA and the outcome indexs s during the follow-up in male patients with axial spondyloarthritis (Multivariable Model Adjusted for Contextual factors ). Table S3. Effects of hyperuricemia on the outcome indexs during the fllow-up in male patients with axial spondyloarthritis (Multivariable Model Adjusted for Contextual factors ). Table S4. Effects of bDMARD use on the outcome indexs during the fllow-up in male patients with axial spondyloarthritis (Multivariable Model Adjusted for Contextual factors ). Table S5. Effects of cDMARD use on the outcome indexs during the fllow-up in male patients with axial spondyloarthritis (Multivariable Model Adjusted for Contextual factors ). Table S6. Effects of NSAID use on the outcome indexs during the fllow-up in male patients with axial spondyloarthritis (Multivariable Model Adjusted for Contextual factors). [file 12891_2022_5657_MOESM1_ESM.docx]

**Table S1 Longitudinal relationship between serum uric acid and the outcome indexs during the follow-up in male patients with axial spondyloarthritis(Univariable Model)**

| **outcome** | **β(95%CI)** | **P** |
| --- | --- | --- |
| PtGA,0–100 | -1.981(-3.863,-0.099) | 0.039 |
| Spinal pain ,0–100 | -1.978(-3.862,-0.094) | 0.04 |
| Spinal pain in ninght,0–100 | -1.462(-3.022,0.098) | 0.066 |
| BASDAI,0–10 | -0.175(-0.356,0.006) | 0.058 |
| BASFI,0–10 | -0.125(-0.273,0.023) | 0.099 |
| ASDAS‐CRP | -0.096(-0.187,-0.005) | 0.039 |
| SF-36:Physical Functioning(PF) | 2.039(0.58,3.499) | 0.006 |
| SF-36:Role-Physical(RP) | 2.662(-0.577,5.902) | 0.107 |
| SF-36:BP(Bodily Pain） | 1.089(-0.229,2.408) | 0.105 |
| SF-36:General Health(GH) | 2.577(0.182,4.972) | 0.035 |
| SF-36:Vitality(VT) | 0.596(-0.582,1.773) | 0.321 |
| SF-36:Social Functioning(SF) | 2.267(0.705,3.83) | 0.004 |
| SF-36:Role-Emotional(RE) | 0.668(-2.949,4.285) | 0.717 |
| SF-36:Mental Health(MH) | 0.419(-0.666,1.504) | 0.449 |
| CRP(mg/liter) | -0.385(-0.989,0.219) | 0.211 |
| ESR(mm/h) | -0.001(-0.657,0.655) | 0.997 |

**Table S2 Longitudinal relationship between SUA and the outcome indexs s during the follow-up in male patients with axial spondyloarthritis(Multivariable Model Adjusted for Contextual factors )**

| outcome | β(95%CI) | *p* |
| --- | --- | --- |
| PtGA,0–100 | -2.059(-4.032,-0.086) | 0.041 |
| Spinal pain ,0–100 | -1.836(-3.899,0.227) | 0.081 |
| Spinal pain in ninght,0–100 | -1.307(-3.111,0.498) | 0.156 |
| BASDAI,0–10 | -0.156(-0.34,0.028) | 0.096 |
| BASFI,0–10 | -0.044(-0.174,0.086) | 0.509 |
| ASDAS‐CRP | -0.075(-0.169,0.018) | 0.115 |
| SF-36:Physical Functioning(PF) | 1.191(-0.266,2.648) | 0.109 |
| SF-36:Role-Physical(RP) | 3.644(-0.114,7.403) | 0.057 |
| SF-36:Bodily Pain(BP) | 1.442(-0.371,3.255) | 0.119 |
| SF-36:General Health(GH) | 1.549(-1.554,4.652) | 0.328 |
| SF-36:Vitality(VT) | 1.751(0.415,3.087) | 0.01 |
| SF-36:Social Functioning(SF) | 2.968(1.067,4.869) | 0.002 |
| SF-36:Role-Emotional(RE) | 2.421(-1.747,6.589) | 0.255 |
| SF-36:Mental Health(MH) | 0.834(-0.608,2.275) | 0.257 |
| CRP(mg/liter) | -0.259(-1.017,0.5) | 0.504 |
| ESR(mm/h) | 0.319(-0.552,1.191) | 0.472 |

**Table S3 Effects of hyperuricemia on the outcome indexs during the fllow-up in male patients with axial spondyloarthritis(**Multivariable Model Adjusted for **Contextual factors )**

| outcome | β(95%CI) | *p* |
| --- | --- | --- |
| PtGA,0–100 | -1.482(-7.136,4.172) | 0.607 |
| Spinal pain ,0–100 | -1.06(-6.602,4.481) | 0.708 |
| Spinal pain in ninght,0–100 | -1.187(-6.92,4.546) | 0.685 |
| BASDAI,0–10 | -0.024(-0.523,0.475) | 0.926 |
| BASFI,0–10 | -0.062(-0.514,0.389) | 0.786 |
| ASDAS‐CRP | -0.087(-0.341,0.166) | 0.499 |
| SF-36:Physical Functioning(PF) | 2.858(-4.563,10.279) | 0.45 |
| SF-36:Role-Physical(RP) | 9.094(-6.834,25.021) | 0.263 |
| SF-36:BP(Bodily Pain） | 1.542(-4.254,7.338) | 0.602 |
| SF-36:General Health(GH) | 3.961(-7.549,15.471) | 0.5 |
| SF-36:Vitality(VT) | 4.854(-1.635,11.342) | 0.143 |
| SF-36:Social Functioning(SF) | 4.139(-5.614,13.893) | 0.406 |
| SF-36:Role-Emotional(RE) | 4.545(-10.951,20.041) | 0.565 |
| SF-36:Mental Health(MH) | 2.571(-3.95,9.091) | 0.44 |
| CRP(mg/liter) | -0.779(-3.622,2.064) | 0.591 |
| ESR(mm/h) | -0.686(-3.938,2.566) | 0.679 |

**Table S4 Effects of bDMARD use on the outcome indexs during the fllow-up in male patients with axial spondyloarthritis(**Multivariable Model Adjusted for **Contextual factors )**

| outcome | β(95%CI) | *p* |
| --- | --- | --- |
| PtGA,0–100 | 1.148(-3.325,5.622) | 0.615 |
| Spinal pain ,0–100 | -0.547(-5.108,4.014) | 0.814 |
| Spinal pain in ninght,0–100 | 0.489(-4.465,5.443) | 0.847 |
| BASDAI,0–10 | -0.078(-0.516,0.36) | 0.728 |
| BASFI,0–10 | -0.072(-0.446,0.302) | 0.706 |
| ASDAS‐CRP | 0.222(0.007,0.436) | 0.043 |
| SF-36:Physical Functioning(PF) | 2.285(-3.644,8.214) | 0.45 |
| SF-36:Role-Physical(RP) | 4.273(-8.839,17.385) | 0.523 |
| SF-36:BP(Bodily Pain） | 1.157(-3.215,5.529) | 0.604 |
| SF-36:General Health(GH) | 0.889(-7.619,9.397) | 0.838 |
| SF-36:Vitality(VT) | 5.305(0.882,9.728) | 0.019 |
| SF-36:Social Functioning(SF) | 7.977(0.469,15.484) | 0.037 |
| SF-36:Role-Emotional(RE) | 11.701(0.56,22.841) | 0.04 |
| SF-36:Mental Health(MH) | 2.775(-1.354,6.904) | 0.188 |
| CRP(mg/liter) | 3.436(1.407,5.465) | 0.001 |
| ESR(mm/h) | 2.181(-0.332,4.694) | 0.089 |

**Table S5 Effects of cDMARD use on the outcome indexs during the fllow-up in male patients with axial spondyloarthritis(**Multivariable Model Adjusted for **Contextual factors )**

| outcome | β(95%CI) | *p* |
| --- | --- | --- |
| PtGA,0–100 | 1.54(-3.45,6.53) | 0.545 |
| Spinal pain ,0–100 | 1.146(-3.732,6.025) | 0.645 |
| Spinal pain in ninght,0–100 | -0.654(-5.445,4.136) | 0.789 |
| BASDAI,0–10 | 0.027(-0.355,0.409) | 0.89 |
| BASFI,0–10 | -0.212(-0.483,0.059) | 0.124 |
| ASDAS‐CRP | -0.138(-0.351,0.074) | 0.202 |
| SF-36:Physical Functioning(PF) | 1.407(-2.857,5.672) | 0.518 |
| SF-36:Role-Physical(RP) | -13.936(-25.015,-2.856) | 0.014 |
| SF-36:BP(Bodily Pain） | -4.065(-9.107,0.977) | 0.114 |
| SF-36:General Health(GH) | -0.077(-7.159,7.005) | 0.983 |
| SF-36:Vitality(VT) | -2.142(-5.645,1.361) | 0.231 |
| SF-36:Social Functioning(SF) | -8.383(-15.261,-1.505) | 0.017 |
| SF-36:Role-Emotional(RE) | -25.885(-39.137,-12.633) | <0.001 |
| SF-36:Mental Health(MH) | -0.913(-4.607,2.781) | 0.628 |
| CRP(mg/liter) | -1.978(-3.572,-0.384) | 0.015 |
| ESR(mm/h) | 0.04(-2.512,2.593) | 0.975 |

**Table S6 Effects of NSAID use on the outcome indexs during the fllow-up in male patients with axial spondyloarthritis(**Multivariable Model Adjusted for **Contextual factors )**

| outcome | β(95%CI) | *p* |
| --- | --- | --- |
| PtGA,0–100 | 2.6551(-7.688,2.719) | 0.349 |
| Spinal pain ,0–100 | 2.5159(-8.743,1.119) | 0.13 |
| Spinal pain in ninght,0–100 | 2.3608(-6.162,3.092) | 0.516 |
| BASDAI,0–10 | 0.1996(-0.493,0.29) | 0.611 |
| BASFI,0–10 | 0.1535(-0.294,0.307) | 0.967 |
| ASDAS‐CRP | 0.1166(-0.287,0.17) | 0.617 |
| SF-36:Physical Functioning(PF) | 2.2538(-3.927,4.907) | 0.828 |
| SF-36:Role-Physical(RP) | 5.9144(-1.906,21.278) | 0.101 |
| SF-36:BP(Bodily Pain） | 2.5346(-0.207,9.729) | 0.06 |
| SF-36:General Health(GH) | 3.8559(-2.071,13.043) | 0.155 |
| SF-36:Vitality(VT) | 2.083(1.669,9.834) | 0.006 |
| SF-36:Social Functioning(SF) | 3.9418(2.23,17.681) | 0.012 |
| SF-36:Role-Emotional(RE) | 6.3739(4.443,29.428) | 0.008 |
| SF-36:Mental Health(MH) | 2.2747(-2.419,6.498) | 0.37 |
| CRP(mg/liter) | 0.8906(-0.805,2.686) | 0.291 |
| ESR(mm/h) | 1.1663(-3.603,0.969) | 0.259 |
